# Rhizosphere Microbial Community Structure Is Selected by Habitat but Not Plant Species in Two Tropical Seagrass Beds

**DOI:** 10.3389/fmicb.2020.00161

**Published:** 2020-03-04

**Authors:** Xia Zhang, Chunyu Zhao, Shuo Yu, Zhijian Jiang, Songlin Liu, Yunchao Wu, Xiaoping Huang

**Affiliations:** ^1^Key Laboratory of Tropical Marine Bio-resources and Ecology, South China Sea Institute of Oceanology, Chinese Academy of Sciences, Guangzhou, China; ^2^Innovation Academy of South China Sea Ecology and Environmental Engineering, Chinese Academy of Sciences, Guangzhou, China; ^3^University of Chinese Academy of Sciences, Chinese Academy of Sciences, Beijing, China

**Keywords:** seagrass bed, rhizosphere, microbial community, habitat, patch type

## Abstract

Rhizosphere bacterial community structures and their determining drivers have been studied in a variety of marine and freshwater ecosystems for a range of plant species. However, there is still limited information about the influence of habitat on microbial communities in seagrass beds. This study aimed to determine which factors (habitat and plant species) have crucial roles on the rhizospheric bacteria associated with two tropical seagrass species (*Thalassia hemprichii* and *Enhalus acoroides*) that are dominant at Xincun Bay and Tanmen Harbor in Hainan Island, South China. Using Illumina HiSeq sequencing, we observed substantial differences in the bacterial richness, diversity, and relative abundances of taxa between the two habitats, which were characterized differently in sediment type and nutrient status. Rhizospheric bacteria from sandy sediment at the eutrophic Xincun Bay were dominated by Desulfobacteraceae and Helicobacteraceae, which are primarily involved in sulfate cycling, whereas rhizosphere microbes from the reef flat at oligotrophic Tanmen Harbor were dominated by Vibrionaceae and Woeseiaceae, which may play important roles in nitrogen and carbon fixing. Additionally, we speculated that host-specific effects of these two seagrass species may be covered under nutrient-rich conditions and in mixed community patches, emphasizing the importance of the nutrient status of the sediment and vegetation composition of the patches. In addition, our study confirmed that Proteobacteria was more adapted to the rhizosphere environment than to low-carbon conditions that occurred in bulk sediment, which was primarily dominated by well-known fermentative bacteria in the phylum Firmicutes.

## Introduction

Sediment, especially the rhizosphere of plants, is a complex and heterogeneous hotspot inhabited by various microorganisms (Wei et al., 2017). Lennon and Jones (2011) noted that the physicochemical properties of the soil, together with plant species, dominated which members of microorganisms can grow and thrive. A large number of studies have suggested that the host plant plays a major determining role in structuring the composition of its rhizosphere microbiota (Aleklett et al., 2015; Behera et al., 2017). The popular notion indicated that this selective effect of a host plant is a direct result of plant–microbe interactions due to the unique composition of root exudates. In addition, other studies have demonstrated that the host plant and sediment are both major factors affecting community structure under natural conditions (Berg and Smalla, 2009; Tkacz et al., 2015). In some cases, sediment can override the plant effects on rhizosphere microorganisms, although it is unclear which sediment properties (i.e., pH, texture, and nutrient status) contribute to this phenomenon (Nunan et al., 2005; Veresoglou et al., 2011). Singh et al. (2007) suggested that rhizosphere bacterial communities are influenced by similar environmental factors, indicating that different plant species do not select specific microbial populations in their rhizospheres. Similarly, Veresoglou et al. (2011) revealed that nutrient fertilization of soil may also mask the ability of plant species to shape their own rhizosphere microbial community.

A great deal of studies has focused on investigating specific root colonizers in rhizosphere sediments (Walker et al., 2003; Bais et al., 2006). Members of the phylum Proteobacteria commonly respond positively to plant roots (Bulgarelli et al., 2012; Peiffer et al., 2013), as they can utilize a variety of low-molecular-weight substrates as electron accepters, which are abundant in plant root exudates (Goldfarb et al., 2011; Behera et al., 2017). Furthermore, on account of seagrasses growing in largely anoxic, reduced marine sediments (Borum et al., 2005) that are often enriched in the potent phytotoxin H_2_S, hence, the rhizosphere of these plants is dominated by bacteria involved in the sulfur cycle (Jensen et al., 2007; Cúcio et al., 2016). In particular, Jensen et al. (2007) showed that sulfate-reducing bacteria (SRBs) and sulfide-oxidizing bacteria (SOBs) are located in different microniches within the seagrass rhizosphere and that some SOBs were considered to benefit from the localized production of sulfide by root-associated SRBs (Hines et al., 1999; Thomas et al., 2014).

In natural environments, co-occurring plants in mixed communities may interact with other plants when their roots grow intimately, and the exudates from one species can influence the root colonizers of neighboring hosts (Kennedy et al., 2005; Bezemer et al., 2006). Nunan et al. (2005) observed little or no effect of plant species on the composition of the rhizosphere bacterial community when comparing five co-occurring species from natural grassland. However, Aleklett et al. (2015) showed that root bacterial communities differed significantly among three plant species, even though they grew in close proximity to each other. Thus, the influences of mixed plant communities may be more complex than the effects of a single plant species on rhizosphere bacterial communities.

Although the rhizosphere microbiome of terrestrial plants in mixed stands has been well studied, limited information is available for seagrasses. *Thalassia hemprichii* and *Enhalus acoroides* are two dominant species in seagrass beds at Xincun Bay and Tanmen Harbor. In this study, for the first time, we used high-resolution amplicon sequencing of the bacterial 16S rRNA gene to characterize the rhizosphere microbiome of these two tropical seagrass species from Hainan Island, South China. We hypothesized that there would be a diverse microbial community composition of the rhizospheres of different host species and habitats. In addition, we also assessed whether rhizosphere compartments are species-dependent when these two seagrasses grow in a mixed community.

## Materials and Methods

### Study Area

**FIGURE 1:**
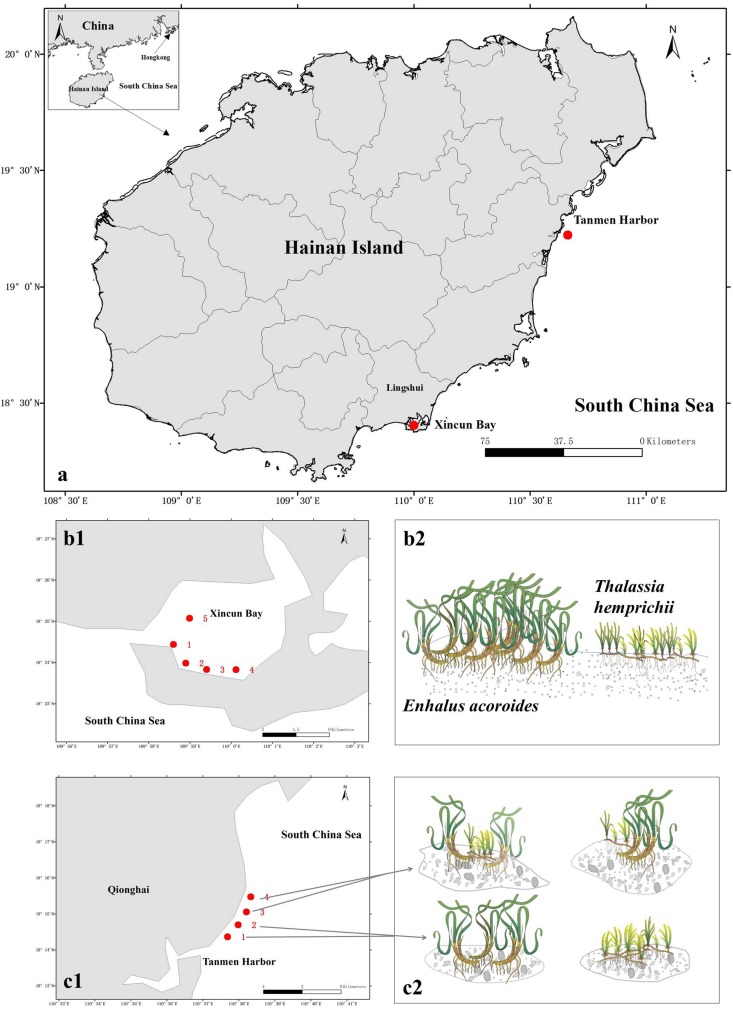
**(a)** Map of sampled seagrass beds in Hainan Island, South China Sea. **(b1,b2)** Sampling stations and vegetation compositions of seagrass beds at Xincun Bay. *Enhalus acoroides* and *Thalassia hemprichii* grow continuously and independently cover a large area. **(c1,c2)** Sampling stations and patch types at Tanmen Harbor. These two species grow in scattered patches forming single-species populations at sites 1 and 2 and mixed communities at sites 3 and 4. Diagram produced using the Integration and Application Network (IAN), University of Maryland Center for Environmental Sciences, Cambridge, Maryland.

The two study areas (Figure 1A) are located at Xincun Bay (18°24′34^″^ N, 109°57′42^″^ E) and Tanmen Harbor (19°15′12^″^ N, 110°38′04^″^E) on the east coast of Hainan Island. At Xincun Bay, the two seagrass species grow densely and continuously along the southern coast, whereas on the west coast, the meadows cover a smaller area. Five sampling sites were surveyed across the both coasts at Xincun Bay (Figure 1B). At Tanmen Harbor, these two species form two types of communities in separate scattered patches, one of which is composed of single-species population (represented by sites 1 and 2; Figures 1C1,C2), whereas the other is composed of two co-occurring species in the same patch (represented by sites 3 and 4; Figures 1C1,C2). The distance between each sampling sites at Xincun Bay and Tanmen Harbor was approximately 0.5 to 1.5, and 1.0 km, respectively. The substrate of the two habitats was sandy sediment at Xincun Bay and coral reef flat at Tanmen Harbor. Bulk and vegetated sediments were sampled in the summer of 2016. Two sets of bulk sediments were also collected at two locations, one in the surroundings of *E. acoroides* (Bulk1) and another in the surroundings of the *T. hemprichii* (Bulk2).

### Sampling Strategy and DNA Extraction

Seagrass and bulk sediments were collected with a custom-made corer (100-cm length, 10-cm inner diameter) during the low-tide period, when the water depth was approximately 20 to 50 cm. Sediment cores were taken to a depth of maximum penetration 20 cm for *E. acoroides*, 10 cm for *T. hemprichii*, and 10 cm for bulk sediment, respectively. Five to ten cores of each seagrass species were randomly collected at each sampling site. To obtain the rhizosphere from the seagrasses, we adapted a method that is commonly used for the retrieval of rhizospheres from terrestrial plants (Costa et al., 2006; Lundberg et al., 2012). Briefly, each seagrass core was slowly emptied in a tray, maintaining the intact structure of the sectioned sediment. Roots from the selected seagrasses were manually shaken to remove loose sediment, and the sediment that remained attached to the roots (rhizosphere) was collected for further analysis. Each rhizosphere sample was a pool of sediment from five cores. The samples were transported to the laboratory in a cool box and immediately processed on arrival. For the analysis of bacterial diversity, 0.5 g of the sediment sample (fresh weight) was used to extract DNA with a PowerSoil DNA kit (Mo Bio Laboratories Inc., Carlsbad, CA, United States) according to the manufacturer’s protocol.

We collected the residual sediment without seagrass roots to obtain pore water samples. Sediment pore water was sampled using ultrapure rinsed microporous (0.45-μm membrane) polymer tube samplers inserted vertically into the sediment. Pore water suction was performed with an acid-washed 60-mL syringe set into a depression, and pore water was acidified immediately after sampling with 0.5% vol/vol HCl. The concentrations of ammonium (NH_4_-N), nitrate (NO_3_-N), nitrite (NO_2_-N), and phosphate (PO_4_-P) in the pore water samples were determined via a colorimetric method using a Lachat QC8500 Flow Injection Autoanalyzer (Lachat Instruments, Loveland, CO, United States). The particle sizes of the sediment samples were analyzed using a laser diffractometer (Malvern Mastersizer, 2000, Malvern Panalytical Ltd., Malvern, United Kingdom) capable of analyzing particle sizes between 0.02 and 2000 μm.

### Polymerase Chain Reaction Amplification

Using the polymerase chain reaction primers F515 (GTGCCAGCMGCCGCGG) and R806 (GGACTACHVGGG TWTCTAAT), the bacterial V4 hypervariable region of the 16S ribosomal RNA gene was amplified (Caporaso et al., 2012). The primer set was modified to include (Illumina, San Diego, CA, United States) adapters and barcode sequences using a dual indexing approach. Raw reads were obtained using an Illumina HiSeq 2500 PE250 platform (Illumina, San Diego, CA, United States). The obtained sequences were analyzed using the QIIME version 1.9.1 pipeline (Caporaso et al., 2010b). Paired-end reads were merged into full-length amplicon sequences with FLASH v1.2.7 (Magoč and Salzberg, 2010). Chimeric sequences were discarded based on prediction by UCHIME algorithm using the Gold reference database (Edgar et al., 2011). The effective sequences were clustered into OTUs (operational taxonomic units) using Uparse at a threshold of 97% similarity. The resulting representative sequences set were aligned, using PyNAST (Caporaso et al., 2010a) and given a taxonomic classification, using Ribosomal Database Project (RDP) (Wang et al., 2007), which was retrained with the Greengenes v13.5 (McDonald et al., 2012) with a minimum confidence of 0.8.

Raw data files in FASTQ format were deposited in the NCBI Sequence Read Archive with the study accession numbers SRR10723724–SRR10723745 under Bioproject number PRJNA595452.

### Bacterial Community Analysis

Data visualization was performed exclusively in R, and statistical analyses were performed using a combination of QIIME scripts and R (R Core Team, 2016). We were interested in assessing if there were significant differences existing in the α-diversity of the microbial communities associated with different habitats (Xincun Bay vs. Tanmen Harbor) and host plants (*E. acoroides* vs. *T. hemprichii*). To this end, we calculated the Shannon, Chao1, and observed OTUs diversity indices in R. One-way analysis of variance (ANOVA) was used to test the significance among groups for these indices, after which Tukey *post hoc* test was applied to test the pairwise group differences. The community composition between groups (β-diversity) was assessed using Bray-Curtis similarity matrices in PRIMER (v7; PRIMER-e, Plymouth, United Kingdom). These dissimilarities were then visualized using cluster analysis and principal coordinate analysis (PCoA) methods.

Two-way permutational multivariate ANOVA (PERMANOVA) was performed to identify the overall compositional differences between the habitat and seagrass species with a significance level of *p* < 0.01, based on a Euclidean distance similarity matrix that was also calculated in PRIMER. For each habitat, a one-way PERMANOVA was applied to identify differences between sediments (rhizosphere vs. bulk), patches (single vs. mixed), and seagrass species. Subsequently, the similarity percentage method (SIMPER, Clarke, 1993) was used to assess which taxa were responsible for the differences observed between the groups.

## Results

### Sediment Features

With respect to the physical properties of the sediment, grain size or texture of the sediment between the two habitats was completely different. The sediment was coarse-gravel sandy at Tanmen Harbor and finer-grained sandy at Xincun Bay (Table 1). Dissolved inorganic nitrogen (DIN) and dissolved inorganic phosphate (DIP) concentrations in pore water at Xincun Bay (322.8 ± 135.7 and 8.5 ± 6.1 μmol/L, respectively) were much higher than those observed for the Tanmen Harbor sediment (90.9 ± 42.1 and 0.4 ± 0.6 μmol/L, respectively). Furthermore, both DIN and DIP contents were much higher in vegetated sediment than those measured in bare sediments (Table 1).

**TABLE 1:** Abiotic characteristics of sediment and pore water at the two habitats, including the rhizosphere and bulk samples.

Sample Name	Sediment				Pore water	
	Gravel (%)	Sand (%)	Silt (%)	Clay (%)	DIN (μmol/L)	DIP (μmol/L)
XC-1	0	87.8 ± 3.2	11.6 ± 2.9	0.6 ± 0.3	489.8 ± 37.6	13.9 ± 7.7
XC-2	0	91.4 ± 0.1	8.4 ± 0.1	0.2 ± 0.02	332.8 ± 30.1	13.8 ± 9.4
XC-3	0	87.5 ± 2.1	12.1 ± 2.0	0.4 ± 0.1	120.5 ± 25.3	10.9 ± 2.9
XC-4	4.2 ± 4.7	90.8 ± 6.3	4.9 ± 1.6	0.1 ± 0.03	429.4 ± 15.7	2.6 ± 0.2
XC-5	13.0 ± 0.6	83.8 ± 0.8	3.1 ± 0.8	0.1 ± 0.1	336.0 ± 103.5	3.3 ± 0.6
Bulk-1	2.3 ± 3.3	87.2 ± 4.5	10.0 ± 7.4	0.4 ± 0.4	298.6	5.5
Bulk-2	2.7 ± 1.9	85.3 ± 3.9	9.7 ± 5.7	0.3 ± 0.2	158.4	7.7
Average	3.2 ± 4.6	87.7 ± 2.7	8.5 ± 3.4	0.3 ± 0.2	322.8 ± 135.7	8.5 ± 6.1
TM-1	44.9 ± 10.6	50.6 ± 9.8	4.3 ± 0.7	0.2 ± 0.1	91.3 ± 18.6	1.1 ± 1.2
TM-2	37.5 ± 7.3	56.8 ± 4.3	5.3 ± 2.6	0.3 ± 0.4	148.7 ± 17.1	0.1 ± 0.05
TM-3	42.7 ± 14.3	53.7 ± 13.1	3.6 ± 1.1	0.1 ± 0.1	98.3 ± 49.5	0.1 ± 0.05
TM-4	31.6 ± 18.7	63.8 ± 16.7	4.4 ± 1.7	0.2 ± 0.2	79.1 ± 5.8	0.1 ± 0.007
Bulk-1	47.9 ± 4.4	45.4 ± 1.7	5.9 ± 2.1	0.08 ± 0.6	37.7	UDL
Bulk-2	47.3 ± 11.3	49.8 ± 14.1	2.8 ± 2.2	0.1 ± 0.1	36.6	UDL
Average	42.0 ± 6.3	53.4 ± 6.4	4.4 ± 1.1	0.2 ± 0.1	90.9 ± 42.1	0.4 ± 0.6

*XC, Xincun Bay; TM, Tanmen Harbor; UDL, under detection limit.*

### α-Diversity of Bacterial 16S rRNA Gene

**FIGURE 2:**
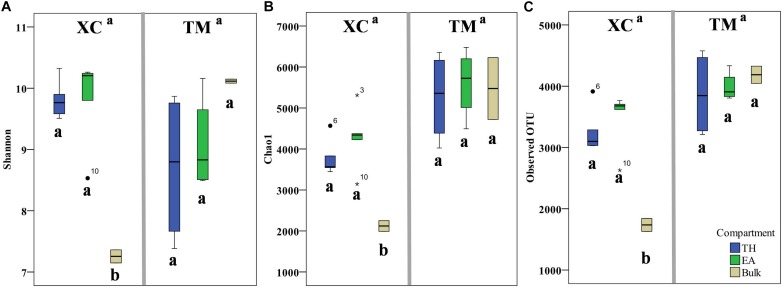
The values of α-diversity indices, Shannon **(A)**, Chao1 **(B)**, and observed number of OTUs **(C)**, are shown as boxplots for the two habitats (Xincun Bay, XC; Tanmen Harbor, TM) for the two seagrass species (*Thalassia hemprichii*, TH; *Enhalus acoroides*, EA). Different letters means significantly different (one-way ANOVA, *p* < 0.05).

A total of 1,154,284 raw reads were obtained from the HiSeq sequencing analysis. After filtering the low-quality reads using the QIIME standard pipeline and trimming the primers, adapters, and barcodes, 1,105,980 qualifying clean reads were clustered into 9567 OTUs using the average neighbor algorithm with a cutoff at 97% similarity. In addition, 93.0% of sequences could be classified at the phylum level, whereas 34.1% of sequences could be classified to the genus level. With respect to α-biodiversity, Shannon index values ranging from 7.15 to 10.32 and 6.87 to 10.20 were observed at Xincun Bay and Tanmen Harbor, respectively, although the differences between the two locations were not significant (one-way ANOVA, *p* = 0.92; Figure 2). Similarly, the values obtained for other indices, including Chao1 and observed OTU numbers, were not significantly different between two habitats (*p* values were 0.08 and 0.13, respectively). At Xincun Bay, higher biodiversities were observed in the rhizosphere samples when compared with those of the bulk sediments (Shannon, *p* = 0.0001; Chao1, *p* = 0.003; observed OTU, *p* = 0.0001; Figure 2), but no significant difference between the two plant species was observed (Shannon, *p* = 0.98; Chao1, *p* = 0.26; observed OTU, *p* = 0.47). At Tanmen Harbor, neither rhizosphere vs. bulk (Shannon, *p* = 0.29; Chao1, *p* = 0.38; observed OTU, *p* = 0.34) nor EA vs. TH differed significantly (Shannon, *p* = 0.41; Chao1, *p* = 0.48; observed OTU, *p* = 0.50).

### Community Composition of Bacterial 16S rRNA Gene

**FIGURE 3:**
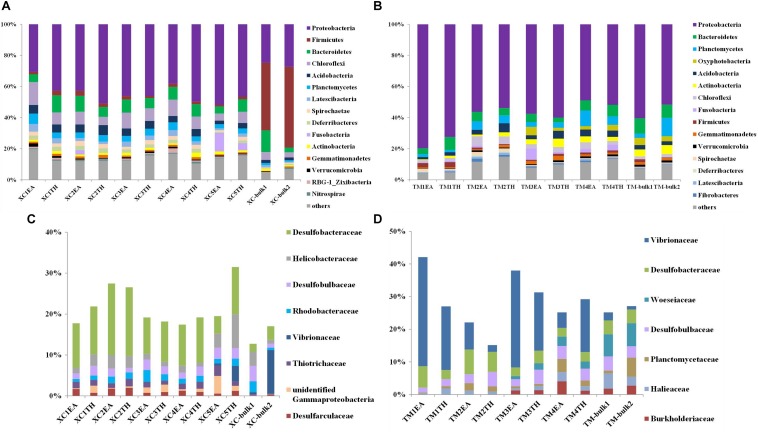
Composition of major bacterial phyla (top 15) and dominant families of Proteobacteria (relative abundance >1%) in the bulk and rhizosphere sediments of EA and TH samples at Xincun Bay **(A,C)** and Tanmen Harbor **(B,D)**. The abbreviations are the same as those used in Figure 2.

Figures 3A,B show the relative abundance of the top 15 bacterial phyla detected in the two seagrass habitats. At Xincun Bay, the most abundant phylum was Proteobacteria (41.5% ± 9.2%), followed by Firmicutes (9.6% ± 17.9%), Bacteroidetes (7.8% ± 3.0%), Chloroflexi (7.7% ± 3.1%), and Acidobacteria (4.2% ± 1.5%). In rhizosphere sediments, the dominant phyla (relative abundance >5%) included Proteobacteria (44.5% ± 6.4%), Chloroflexi (8.4% ± 2.9%), and Bacteroidetes (7.6% ± 2.1%) (Figure 3A). At the family level, the dominant groups (relative abundance >1%) affiliated with Proteobacteria in the rhizosphere at Xincun Bay included Desulfobacteraceae, Helicobacteraceae, and Desulfobulbaceae (Figure 3C). Different from rhizosphere sediments, the phylum Firmicutes (47.7% ± 5.9%) was greatly enhanced in bulk sediments (Figure 3C). At Tanmen Harbor, the most abundant phylum was Proteobacteria (59.3% ± 9.7%), followed by Bacteroidetes (6.4% ± 2.2%), Planctomycetes (5.1% ± 3.4%), Oxyphotobacteria (3.5% ± 1.3%), and Acidobacteria (3.4% ± 1.4%) (Figure 3B). Within the phylum Proteobacteria, the families Vibrionaceae, Woeseiaceae, Desulfobacteraceae, and Desulfobulbaceae comprised 23.3% of the total 16S rRNA sequences (Figure 3D), and the dominant genera in Vibrionaceae were *Vibrio* and *Photobacterium*.

### Comparison of Bacterial Community Structure Among Sample Types

**FIGURE 4:**
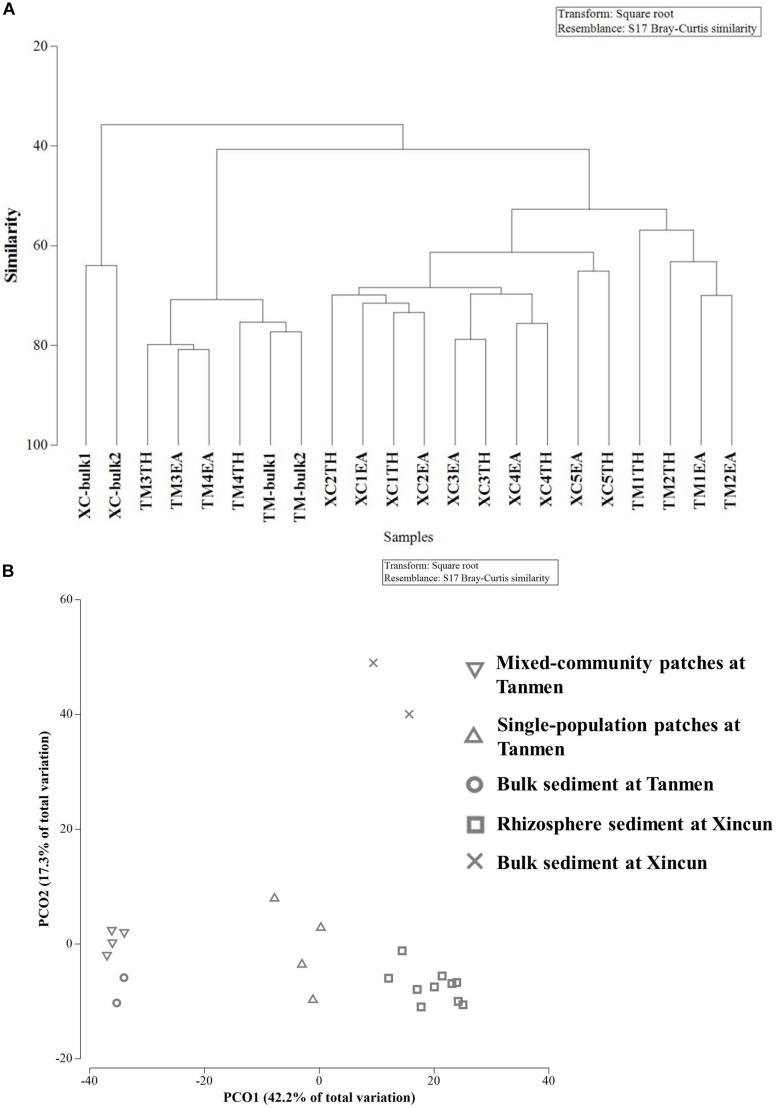
Cluster **(A)** and principal coordinates analysis **(B)** of bacterial communities at the genus level based on the Bray-Curtis distance.

Cluster and PCoA (Figure 4) analyses at the genus level showed a clear distinction between the two habitats. Among the Xincun Bay samples, although two plant species grew independently, their rhizosphere microbial compositions were tightly clustered, and the largest difference was between the rhizosphere and bulk sediment. In contrast, at Tanmen Harbor, the rhizosphere microbial communities of the single and mixed patches were distinct. Permutational multivariate ANOVA analysis at the same level also confirmed the existence of significant differences between the groups, that is, Xincun Bay vs. Tanmen Harbor (pseudo-*F* = 10.30, *p* < 0.01), rhizosphere vs. bulk sediment at Xincun (pseudo-*F* = 13.40, *p* < 0.05), and mixed community vs. single population at Tanmen Harbor (pseudo-*F* = 7.00, *p* < 0.05; Table 2).

**TABLE 2:** Permutational multivariate ANOVA results of the assemblage abundance structure between different groups (Pseudo-*F* values and significance level, *p*).

Groups	Pseudo-*F*	SIMPER			
		**Average dissimilarity (%)**	**Contribution of discriminating species**	**Abundance**	**Contribution (%)**

Habitat (XC vs. TM)	10.30 (*p* = 0.001)	64.20	*Vibrio*	2.86, 0.58	*5.17*
			*Photobacterium*	1.52, 0.45	*2.74*
			*Woeseia*	1.10, 0.00	*2.25*
			*Spirochaeta2*	0.40, 1.25	1.94
			Sva0081 sediment group	0.53, 1.23	1.86
			*Propionigenium*	0.31, 0.91	1.77
Species (EA vs. TH)	0.39 (*p* = 0.94)				
Habitat × species	0.67 (*p* = 0.69)				
XC					
Sediment (bulk vs. rhizo)	13.40 (*p* = 0.014)	57.20	*Tepidibacter*	0.17, 3.19	7.96
			*Clostridium* sensu stricto1	0.11, 2.36	5.90
			Unidentified *Clostridium*	0.14, 1.74	4.23
			*Propionigenium*	1.07, 0.15	*2.34*
			Sva0081	1.35, 0.65	*1.89*
			*Spirochaeta2*	1.36, 0.72	*1.70*
Species (EA vs. TH)	0.41 (*p* = 0.93)	24.44			
TM					
Sediment (bulk vs. rhizo)	2.49 (*p* = 0.07)	49.56			
Species (EA vs. TH)	0.50 (*p* = 0.64)	46.24			
Patch (mixed vs. single)	7.00 (*p* = 0.03)	66.02	Unidentified Oxyphotobacteria	1.77, 0.00	3.27
			*Vibrio*	3.33, 3.29	2.84
			*Woeseia*	1.34, 0.00	2.46
			Sva0081	0.00, 1.32	*2.44*
			*Pseudoalteromonas*	0.30, 1.43	*2.03*
			*Spirochaeta2*	0.00, 0.99	*1.84*

*SIMPER results showing the average dissimilarities (%), the greatest contributors to the dissimilarity, and the contribution ratios to the average dissimilarities between the groups. XC, Xincun Bay; TM, Tanmen Harbor; Rhizo, rhizosphere; TH, *Thalassia hemprichii*; EA, *Enhalus acoroides* The normal numbers mean the relative abundance in the former group is higher than that in the latter group; Italicized numbers mean the opposite situation.*

The SIMPER procedure was used to identify the most represented taxa responsible for the dissimilarities between groups (Table 2). For Xincun Bay and Tanmen Harbor groups, *Vibrio*, *Photobacterium*, and *Woeseia* accounted for the largest proportion of the dissimilarity and were primarily enriched at Tanmen Harbor, whereas the abundances of Sva0081, *Spirochaeta2*, and *Propionigenium* were enhanced at Xincun Bay. Furthermore, members of the class Clostridia (Firmicutes) including *Tepidibacter*, *Clostridium* sensu stricto1, and unidentified *Clostridium* accounted for the greatest proportion of the difference between rhizosphere and bulk sediments at Xincun Bay and showed higher abundances in bulk sediment than that observed in the rhizosphere. At Tanmen Harbor, unidentified Oxyphotobacteria, *Vibrio*, *Woeseia*, Sva0081, *Pseudoalteromonas*, and *Spirochaeta2* were identified as the most discriminating taxa of the rhizosphere between different vegetations (mixed community vs. single-species patch).

## Discussion

The chemical composition of root exudates released into the ambient environment is believed to distinguish the rhizosphere from bulk sediment (Peiffer et al., 2013; Ettinger et al., 2017). As shown by the cluster and PCoA results, we observed distinct differences in the microbial composition between the rhizosphere and bulk sediments at Xincun Bay. The bulk sediments were primarily dominated by Firmicutes, which represented 47.7% of all 16S rRNA gene sequences. Generally, bulk sediments are characterized by lower organic matter contents compared to seagrass beds, and the bacterial community may tend to be dominated by the spore-forming Firmicutes, as they are better adapted to carbon-poor environments (Singh et al., 2007). The most represented class of Firmicutes in our investigation was Clostridia, and the SIMPER analysis results showed a relatively high abundance of Clostridia (including *Tepidibacter*, *Clostridium* sensu stricto1, and *Paraclostridium*) in bulk sediments. It has been reported that Clostridia are probably responsible for the anaerobic decomposition of organic matter. Lu et al. (2006) revealed that the bacterial community on rice roots changed from Clostridia as dominant members at 45 days to Comamonadaceae at 90 days. They considered that the impacts of plant growth on release of O_2_ and organic substances are probably the main reason for the dynamic changes of root communities. Based on their results, we speculated that Clostridia may adapt well in more anaerobic environments in comparison to rhizosphere sediments.

Proteobacteria has been recognized as the most abundant phylum and is consistently enriched in the rhizosphere of seagrass compared to bulk sediment (Cúcio et al., 2016; Ettinger et al., 2017; Behera et al., 2017). Proteobacteria members are well-known root colonizers and respond positively to low-molecular-weight substrates, which are abundant in root exudates (Fierer et al., 2007; Goldfarb et al., 2011). The results of our study also revealed that Proteobacteria were well adapted in two seagrass beds in both sampling locations despite their compositions being rather different.

Our results revealed that *Vibrio* and *Photobacterium* were the most abundant genera at the sediments of Tanmen Harbor, which was characterized by relatively low N and P contents. Numerous *Vibrio* and *Photobacterium* strains have been identified as diazotrophs in the rhizosphere of *Zostera marina* (Shieh et al., 1987), *Thalassia testudinum*, and *Syringodium filiforme* (Bagwell et al., 2002), which grew on oligotrophic, carbonated substrates as well. Thus, we considered that biological N_2_ fixation processing below ground could also represent an alternative nutrient supply if the seagrass grew under nutrient-limited conditions (Charpy-Roubaud et al., 2001; Scanlon and Post, 2008; Olson et al., 2009). Plant-driven modification of the rhizosphere microhabitat has been suggested to support mutualistic relationships between seagrasses and diazotrophic bacteria based on reciprocal nutrient exchange, whereby the seagrass roots provide various carbon sources to the diazotrophs (Hansen et al., 2000; Welsh, 2000; Nielsen et al., 2001). Additionally, we observed an enrichment of Woeseiaceae/JTB255 in the Tanmen Harbor seagrass beds (Figure 3D). The results of recent studies showed that the novel Woeseiaceae/JTB255 group dominated in 13 coastal sediments across Europe and Australia (Dyksma et al., 2016). Members of the JTB255 have the genetic potential for chemolithoautotrophy powered by sulfur or hydrogen oxidation and greatly contribute to carbon assimilation (Middelburg, 2011; Dyksma et al., 2016; Mußmann et al., 2017). Therefore, the possible symbiotic interactions between plants and root colonizers may act as an important nitrogen and carbon acquisition pathway for seagrass in oligotrophic sediments.

With higher organic carbon contents in rhizosphere sediments at Xincun Bay, many Proteobacteria members could be involved in the sulfur cycle and carbon remineralization, such as Desulfobacteraceae, Helicobacteraceae, Desulfobulbaceae, and Rhodobacteraceae, which were highly enriched compared with those observed at Tanmen Harbor and in the bulk sediments (Figures 3C,D). Increased amounts of carbon resources have been considered to be responsible for more abundant Desulfobacteraceae clones and higher SRB diversity (Nemergut et al., 2007; Kondo et al., 2012). In our study, the high prevalence of sulfate reducers, that is, Sva0081 (Desulfobacteraceae), SEEP-SRB1 (Desulfobacteraceae), and *Desulfococcus* (Desulfobulbaceae) were complete-oxidizing SRBs with the ability to completely oxidize a diverse range of organic carbon compounds, allowing them to colonize seagrass beds with high organic compound contents (Jensen et al., 2007; Cúcio et al., 2016). Moreover, the sulfur-oxidizing community at Xincun Bay was primarily represented by the genera *Sulfurovum* (Helicobacteraceae) and *Spirochaeta2* (Spirochaetaceae). Members of the genus *Sulfurovum* are considered to be important players that couple sulfide-oxidation and denitrification processes in marine environments and have been frequently observed in the rhizospheres of marine plants (Gomes et al., 2010; Thomas et al., 2014). Spirochaetes species have been suggested to be typical *K*-strategist anaerobes capable of breaking down refractory compounds, including cellulose and chitin (Dai et al., 2016), during decomposition of seagrass detritus (Trevathan-Tackett et al., 2017).

There are several reports on the involvement of rhizosphere plant growth–promoting rhizobacteria (PGPR), including *Pseudomonas*, *Burkholderia*, *Bacillus*, *Trichoderma*, and *Gliocladium* in improving plant growth and health and enhancing the ability of plants to resist a variety of abiotic and biotic stresses (Compant et al., 2010; Lugtenberg and Kamilova, 2009; Saharan and Nehra, 2011). In our study, the participation of *Vibrio*, *Photobacterium*, and Woeseiaceae/JTB255 in nitrogen and carbon fixation may promote plant survival in the oligotrophic Tanmen Harbor. In contrast, because the reduced sulfur compounds that accumulate in eutrophic sediments are known phytotoxins (Lamers et al., 2012), sulfur-metabolizing microbes (*Sulfurovum* and *Spirochaeta2*) may play important roles in promoting seagrass adaptation in Xincun Bay sediments. However, further studies are needed to understand the function of these groups and to identify whether they represent root-specific PGPR.

It has been hypothesized that a complex interaction between sediment types and plant species affects the microbial community composition, with the strength of each factor varying in different ecosystems (Marschner et al., 2001). Numerous studies have shown that plants are a primary factor influencing rhizosphere bacterial communities through the production of root exudates (Devereux, 2005). Osanai et al. (2012) revealed that root bacterial communities varied significantly between plant species, even when growing in close proximity to each other. However, in our case, although *T. hemprichii* and *E. acoroides* at Xincun Bay were observed to grow independently, their rhizosphere microbial compositions were tightly clustered. Bacterial rhizosphere community compositions from different plant species were similar, indicating a weak influence of plant species on the rhizosphere microbial community structure, which was consistent with the results of Singh et al. (2007). A possible explanation for this finding should be ascribed to the nutrient status of the sediment. Previous observations demonstrated a more pronounced impact of plant species traits on microbial communities under lower nutrient conditions, whereas this effect was limited or not obvious in fertilized environments (Bardgett et al., 1999; Marschner et al., 2001; Veresoglou et al., 2011). Supported by this evidence, we speculated that high level of nutrient in substrate may mask the ability of plant species to shape their own rhizosphere microbial community.

Strikingly, the results of our study revealed that the most distinct difference was observed between independent and mixed patches at Tanmen Harbor. Furthermore, the rhizosphere microbial communities of the two target plant species were clustered together when they were in mixed communities. We observed that these two co-occurring species grew in close proximity to each other above ground and that their root systems were entangled belowground at Tanmen Harbor. Aleklett et al. (2015) indicated that the roots of different species growing intimately with each other may allow for the exudates from one plant species to influence the rhizosphere of neighboring plants. Westover et al. (1997) showed that the composition of rhizobiomes in seminatural grasslands was affected by coexisting plant species. Similarly, Nunan et al. (2005) and Osanai et al. (2012) also observed little or no effect of plant species on the composition of rhizosphere bacterial communities when comparing co-occurring species from natural grassland, suggesting that the highly entangled root systems in a complex plant community prevented the influences of host-specific effects on rhizospheres to be isolated. The observed similarity of rhizospheric bacterial composition of coexisting plants in this study may not be surprising, as bacteria resided in homogenous environments that were primarily shaped by mixed root exudates and decomposing materials. We considered that the lack of significant differences between the rhizosphere microbiomes of the two seagrass species from the mixed community was likely attributed to their interacting root exudates or rhizodeposits, and consequently, the species-specific effect was not obvious.

## Conclusion

In summary, the results of this study provided the evidence that rhizosphere microbes are an essential and active part of the seagrass bed ecosystems. Habitats are shaped by sediment properties have the most important impact on microbial community structure. Some typical diazotrophs (*Vibrio* and *Photobacterium*) and carbon-fixing (Woeseiaceae) taxa were highly enriched at Tanmen Harbor (oligotrophic and carbonated substrate), whereas the abundances of some sulfate reducers (Desulfobacteraceae) and sulfide oxidizers (*Sulfurovum* and *Spirochaeta2*) were observed to be relatively higher at Xincun Bay (eutrophic and sandy sediment). Additionally, the ability of plant species to shape their own rhizosphere microbial community may be modified by the nutrient status of the sediment and the vegetation composition of the patches. Further investigations and culture treatments are required to quantitatively examine the influence of these two factors on bacterial communities in the rhizosphere. In addition, we also observed distinct bacterial compositions between bulk and rhizosphere groups at Xincun Bay, which confirmed the occurrence of a strong “rhizosphere effect.” Bulk sediments were primarily dominated by spore-forming members affiliated with the phylum Firmicutes, which would help them to survive in low-carbon environments, whereas members of the phylum Proteobacteria were more abundant in seagrass beds with higher carbon contents and were primarily involved in sulfur cycle and carbon remineralization.

## Data Availability Statement

The raw data supporting the conclusions of this article will be made available by the authors, without undue reservation, to any qualified researcher.

## Author Contributions

XZ wrote the manuscript. CZ, SY, ZJ, SL, and YW performed the sampling. XH reviewed the manuscript.

## Conflict of Interest

The authors declare that the research was conducted in the absence of any commercial or financial relationships that could be construed as a potential conflict of interest.
